# Nanostructured, mesoporous Au/TiO_2_ model catalysts – structure, stability and catalytic properties

**DOI:** 10.3762/bjnano.2.63

**Published:** 2011-09-15

**Authors:** Matthias Roos, Dominique Böcking, Kwabena Offeh Gyimah, Gabriela Kucerova, Joachim Bansmann, Johannes Biskupek, Ute Kaiser, Nicola Hüsing, R Jürgen Behm

**Affiliations:** 1 Institute of Surface Chemistry and Catalysis, Ulm University, D-89069 Ulm, Germany; 2 Institute of Inorganic Chemistry, Ulm University, D-89069 Ulm, Germany; 3 Transmission Electron Microscopy Group, Ulm University, D-89069 Ulm, Germany; 4 Materials Chemistry, Paris-Lodron University Salzburg, Austria

**Keywords:** Au catalysis, Au/TiO_2_, CO oxidation, gold nanoparticles, model catalysts, thin-film catalyst

## Abstract

Aiming at model systems with close-to-realistic transport properties, we have prepared and studied planar Au/TiO_2_ thin-film model catalysts consisting of a thin mesoporous TiO_2_ film of 200–400 nm thickness with Au nanoparticles, with a mean particle size of ~2 nm diameter, homogeneously distributed therein. The systems were prepared by spin-coating of a mesoporous TiO_2_ film from solutions of ethanolic titanium tetraisopropoxide and Pluronic P123 on planar Si(100) substrates, calcination at 350 °C and subsequent Au loading by a deposition–precipitation procedure, followed by a final calcination step for catalyst activation. The structural and chemical properties of these model systems were characterized by X-ray diffraction (XRD), transmission electron microscopy (TEM), N_2_ adsorption, inductively coupled plasma ionization spectroscopy (ICP–OES) and X-ray photoelectron spectroscopy (XPS). The catalytic properties were evaluated through the oxidation of CO as a test reaction, and reactivities were measured directly above the film with a scanning mass spectrometer. We can demonstrate that the thin-film model catalysts closely resemble dispersed Au/TiO_2_ supported catalysts in their characteristic structural and catalytic properties, and hence can be considered as suitable for catalytic model studies. The linear increase of the catalytic activity with film thickness indicates that transport limitations inside the Au/TiO_2_ film catalyst are negligible, i.e., below the detection limit.

## Introduction

There is a long history of studies in surface science of the elementary steps in catalytic reactions with idealized, planar model systems. In this way, a detailed mechanistic picture, on a molecular scale, has been derived from experimental and theoretical studies for a number of catalytic reactions on metal single crystal surfaces under ultrahigh vacuum (UHV) conditions [1]. It was soon realized, however, that because of the tremendous differences in the materials and reaction conditions between the idealized and realistic cases, the conclusions and results obtained from these model studies could not be easily transferred to the context of a realistic catalytic reaction [2–6]. Here it should be noted that many reactions are not accessible for investigations under surface science conditions, since the rates of specific reaction steps, or of the overall reaction, are too low under these conditions, a classical example of this situation being the synthesis of ammonia [7]. Accordingly, the last two decades saw increasing efforts to bridge the gaps between reaction conditions, often known as the “pressure gap”, and between materials (the “materials gap”) [2–6]. On the one hand, this includes the increasing use of techniques that can also be applied under or close to realistic reaction conditions, in the mbar to 1 bar range, such as high-pressure scanning tunneling microscopy (STM) [8–10], high-pressure X-ray photoelectron spectroscopy (HP-XPS) [11–14], polarization-modulation infrared reflection absorption spectroscopy (PM-IRAS) [15–17] or X-ray absorption spectroscopy (XAS) techniques [18–19], which allow us to gain detailed information on the structure, elemental/molecular chemical composition and electronic/vibrational properties of the catalyst surface and adlayers during reaction. On the other hand, more realistic, but nevertheless structurally well defined model systems were introduced, including in particular planar supported metal catalysts, where metal nanoparticles are supported on thin oxide or other compound films, or on massive oxide substrates [20–22]. These model catalysts were prepared in different ways, e.g., by deposition of the respective active metal phase by evaporation, deposition of preformed metal nanoparticles or chemical impregnation and subsequent activation procedures. While structurally and chemically still reasonably well defined, these systems are also more realistic than pure metal substrates in that they include, e.g., particle size effects or effects resulting from the interface between the support and the active material.

These model systems differ from realistic catalysts, however, in one important aspect with respect to their (internal) transport properties, as given, e.g., by the absence/presence of pore diffusion. Therefore, we recently started to develop a new type of model system, consisting of a nanoscale catalyst layer of a hundred to a few hundred nanometers thickness on a planar support. While both the preparation procedure and the internal surface chemistry and structure closely resemble those of realistic, dispersed catalysts, the transport properties in the nanostructured catalyst are much better controlled. Therefore, these model catalysts should be particularly suited for studies on the influence of the internal nanostructure and transport properties on the reaction characteristics. Furthermore, they may serve also as model systems for the development of catalytically active coatings.

In the following, we present initial results on the preparation, structural and spectroscopic characterization and catalytic properties of ultra-thin Au/TiO_2_ catalyst films, which were prepared by spin-coating a thin film of mesoporous TiO_2_ of 200–400 nm thickness on a flat Si(100) substrate and subsequent loading with Au nanoparticles. After describing the experimental procedures, we first present transmission electron microscopy images and XRD results characterizing the structure and morphology of these films and the distribution and particle size of the Au nanoparticles. The chemical state of the materials was characterized by XPS, and finally the catalytic activity of these model systems was characterized by a scanning mass spectrometer set-up that was modified for these measurements.

## Experimental

### Sample preparation and physical characterization

In a typical procedure, 0.32 g Pluronic^®^ P123 (5 µmol) in 6 g ethanol (0.13 mol) were homogenized with a solution of 2.68 g titanium tetraisopropoxide (9.45 mmol) in 1.36 mL hydrochloric acid (conc.), resulting in a clear TiO_2_ sol. After an aging period of 60 min at room temperature, the sol was spin-coated on the precleaned Si substrates with a spinning speed of 4000 rpm (for 280 nm thickness) for 30 s. To vary the film thickness of the titania films, spinning speeds of 2000 rpm (for 420 nm thickness) and 6000 rpm (for 190 nm thickness) were used instead for 30 s. The Si(100) wafer was cut into small pieces (9 mm × 9 mm) prior to the coating procedure. To remove possible organic contaminants, the wafer was cleaned with acetone, rinsed with distilled water and immersed into a piranha solution (2 H_2_SO_4_ : 1 H_2_O_2_ (30%)) for 5 min, followed by rinsing with water.

Subsequently, the films were aged in air for 8 h at room temperature, followed by drying in an oven (40 °C, 24 h, air). Finally, the structure-directing agent Pluronic^®^ P123 was removed by calcination in air at 350 °C for 3 h, with a ramp rate of 1 K min^−1^.

For the N_2_ sorption and inductively coupled plasma ionization spectroscopy (ICP–OES) measurements, a larger quantity of the titania material was needed: The remaining coating solution was cast in petri dishes and aged analogously to the thin coatings. After the aging step, the material was scraped off and calcined in air for 3 h at 350 °C with a heating ramp rate of 1 K min^−1^.

The Au/TiO_2_/Si catalysts were prepared following a deposition–precipitation (DP) procedure as described previously [23–25]. Up to 5 TiO_2_/Si samples were immersed into 100 mL H_2_O and heated to 60 °C. Then an aqueous solution of 0.01 M HAuCl_4_·3H_2_O was added at constant temperature, while the suspension was stirred and the pH of the solution was kept constant at about 5.5 by dropwise addition of 0.01 M K_2_CO_3_ solution. Subsequently, stirring was continued for additional 30 min, and the solution was then cooled to room temperature. Finally, the Au/TiO_2_/Si precatalysts were washed several times with distilled H_2_O to remove residual potassium and chloride ions as well as unreacted Au species, and then dried at room temperature in vacuum. Prior to the measurements, the Au/TiO_2_ catalyst film was calcined for 1 h at 350 °C in 2 mbar O_2_ (O350 treatment).

The Au/TiO_2_ film thickness was either obtained from the transmission electron microscopy (TEM) measurements (see below) or by AFM profilometry by means of a Topometrix Explorer SPM (scan range: 100 µm) in contact mode. By mechanically removing part of the Au/TiO_2_ film, we generated a free-standing edge of the film on the Si substrate around the sample center, whose height was measured by AFM. Evaluation of single line profiles across the step edge between the bare Si substrate and the region of the intact Au/TiO_2_ film yielded statistically relevant data.

The surface area and the pore diameter of the titania (cast TiO_2_ material, different batches) was determined by N_2_ sorption measurements (Autosorb MP1 and Quadrasorb, Quantachrome). The specific surface area was calculated using the Brunauer–Emmett–Teller (BET) relation in the *p*/*p*_0_ range of 0.05 to 0.3 [26]. The pore size distribution was evaluated from the desorption branch of the isotherms, by the procedure developed by Barrett, Joyner and Halenda (BJH) [27]. XRD measurements were performed on a PANalytical MPD PRO instrument, with Cu Kα radiation (λ = 0.154 nm).

X-ray photoelectron spectroscopy (XPS) measurements were performed using two different XPS systems: In the one system a hemispherical electron analyzer (SPECS, EA 200) was used together with a dual Al/Mg X-ray source (SPECS, RQ 20/38), using Al Kα radiation (1486 eV). Survey spectra were recorded with a pass energy of 197.76 eV, or for detail spectra with a pass energy of 43.95 eV. In the second, we used a PHI 5800 system (Physical Electronics) with a hemispherical electron analyzer in combination with an X-ray source for monochromatic Al Kα radiation. Here, survey spectra were recorded with a pass energy of 93.9 eV (detail spectra with 29.35 eV). ICP–OES measurements were performed on an Ultima 2 instrument (Horiba Jobin Yvon).

### Electron microscopy measurements

The samples were cut into pieces (diamond wire saw) and glued together face-to-face for cross-sectional TEM measurements. These sandwich-like glued sample pieces were mechanical ground, dimpled and polished down to a thickness of <5 µm (Gatan dimple grinder). Low angle (10°) argon ion etching with energies of 5 to 1 keV (Fischione 1010 ion mill) was used to achieve electron transparency with lamella thicknesses of <100 nm. The TEM measurements were carried out on a FEI Titan 80–300 microscope operated at 300 kV in scanning mode (STEM). The microscope was equipped with a high-angle annular dark-field (HAADF) STEM detector (type Fischione). The mass sensitive HAADF contrast (intensity scales with ~*Z*^2^) results in a very strong signal of the Au nanoparticles that could therefore be easily detected and measured by simple thresholding of the STEM images. X-ray spectroscopy to determine the composition was carried out using a Philips CM20 TEM operating at 200 kV equipped with an EDAX energy dispersive X-ray SiLi detector. Scanning electron microscopy was carried out on a Zeiss NVision 040 equipped with in-lens secondary electron detector and back-scatter detector and an EDAX energy dispersive silicon drift X-ray detector. For imaging, a voltage of 1 kV was used, and for EDX spectroscopy an energy of 5 kV was used.

#### CO oxidation activity measurements

The catalytic measurements were performed in a scanning mass spectrometer (SMS) system with a dedicated reaction chamber for reactions at pressures up to several mbar, and a separate second chamber containing a differentially pumped mass spectrometer (for details see [28–29]). The Au/TiO_2_ samples were mounted on a heatable sample stage in the reaction chamber. For the reaction measurements, the reaction chamber was backfilled with the reaction gas mixture (in this case CO and O_2_), with the gas flow being controlled by mass flow controllers (O_2_: Hastings, HFC-302, 0–50 sccm, CO: MKS 1479A, 0–100 sccm).

**Figure 1:**
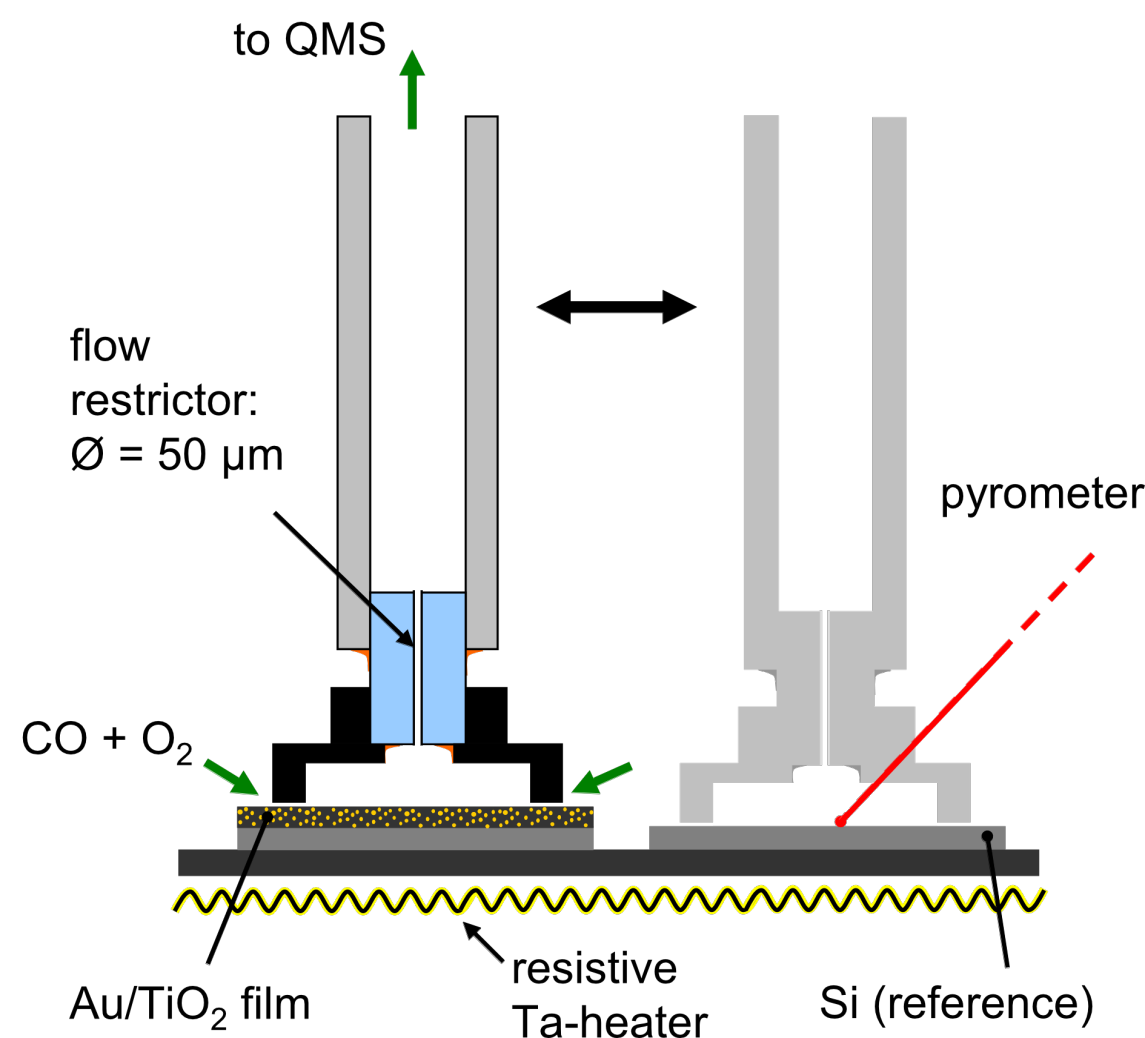
Schematic drawing of the end of the SMS capillary and the sample in the scanning mass spectrometer. Lower part: Sample holder with resistive heater and two separate samples (here: Au/TiO_2_ film on Si and a catalytically inactive Si wafer), upper part: Capillary with flow constriction (can be moved from one sample to the next one).

The reaction chamber is connected to the analysis chamber by a quartz capillary with an inner diameter of 3 mm. At the lower end of the capillary, which reaches into the reaction chamber, a small cylindrical flow restrictor (channel length 3 mm, inner diameter 50 µm) is glued into the slightly widened orifice. The flow restrictor ends in a flat Ti cap (cylindrical volume with inner diameter of 2.5 mm and height of 0.1 mm) to collect the gas species above a defined sample area. In this way, a much larger surface area of the underlying sample contributes to the measured signal than in the previous set-up [28], where the end of the capillary transformed into a tip with a constricted channel (inner diameter: 70 µm, outer diameter: 300 µm). The gas species are then guided towards the analysis chamber and, after leaving the quartz tube, directly into the ion source of a quadrupole mass spectrometer (QMS). A triple-axis, high precision, sample stage allows for free (relative) positioning of the sample underneath the Ti cap, at any lateral position on the sample surface (cf. Figure 1). For the measurements, the pressure in the reaction chamber was varied between 0.5 and 5 mbar, resulting in pressures within the analysis chamber of 1·10^−8^–3·10^−7^ mbar. When the capillary head is fully approached towards the sample surface, we still find no measurable drop in the pressure underneath the Ti cap. Hence, the gas flow into the volume enclosed by the cap is high enough under these conditions that the outgoing flow through the capillary does not lead to a measurable pressure drop in the reaction volume under the Ti cap (cf. [29]). The slow flow of reactants into and out of the reaction volume also results in an accumulation of CO_2_ product gas within the reaction volume, which in turn leads to an enhancement of the CO_2_ signal such that this can be reproducibly detected.

**Figure 2:**
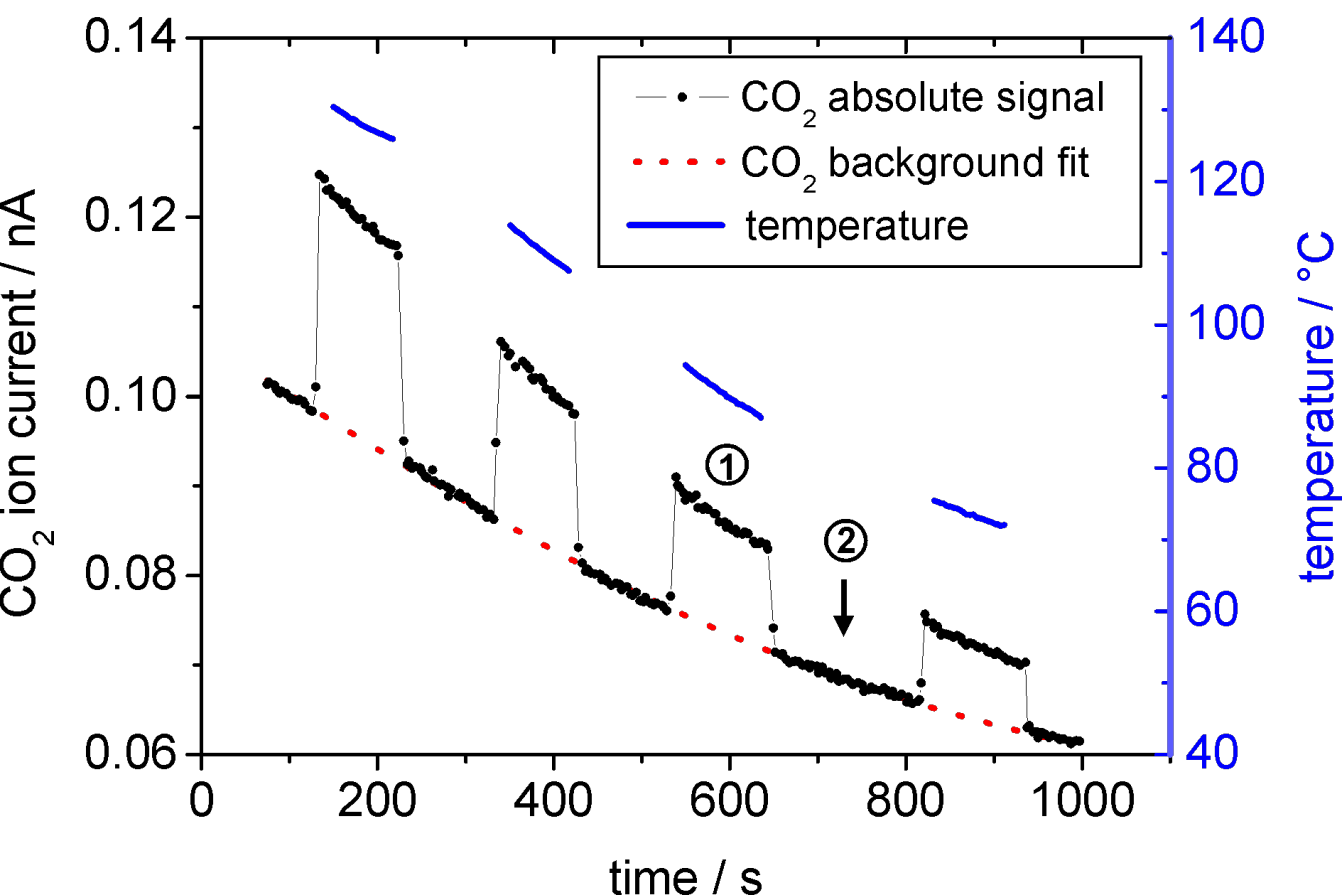
Principle of the SMS measurement on the mesoporous Au/TiO_2_ film with CO oxidation as a test reaction. The CO_2_ signal was measured for decreasing sample temperature (blue data) atop the catalytically active layer (1) and the bare Si reference (2).

To obtain background-corrected product gas concentrations in the reaction measurements, we performed additional measurements on a piece of Si wafer located next to the actual sample, and which served as reference sample. Since the Si surface is catalytically inactive, the CO_2_ concentration above that sample can be considered as a measure of the background signal that is superposed on the CO_2_ concentration arising from the catalytically active sample. This assumption is valid as long as the product gas concentration is low enough to not significantly affect the concentration and flow of the reactant gases (CO and O_2_). Furthermore, the Si reference sample was used for measuring the temperature at the sample surface with a calibrated pyrometer (LumaSense - Impac IPE 140) during the time period when the capillary was located above the Au/TiO_2_ sample (Figure 2). In this way, the temperature measurements are independent of any variations in the specific emissivity of the Au/TiO_2_ films.

CO conversions were calculated from the CO_2_ content in the reaction gas as probed by the mass spectrometer, based on tabulated values for the ionization probability of the respective species CO, O_2_ and CO_2_ [30]. The gas flow into the mass spectrometer was calculated by assuming a maximum difference between the pressures in the reaction chamber and in the reactor (underneath the Ti cap) of 3%. This is justified by the fact that we did not detect any change in the gas flow to the mass spectrometer when approaching the Ti cap towards the sample surface. The incoming CO and O_2_ stream entering the reactor from the outside (from the reaction chamber) was calculated by assuming that all molecules hitting the space between Ti cap and sample surface will enter the reaction volume, and the height of the Ti cap was adjusted relative to the sample surface such that the pressure difference of 3% (see above) was reached. This yields a minimum value for the incoming gas stream, but higher values are possible as well. Accordingly, the conversions given in the next section are maximum values; lower values are also possible.

## Results and Discussion

### Characterization of TiO_2_ coatings and Au/TiO_2_ catalysts

**Figure 3:**
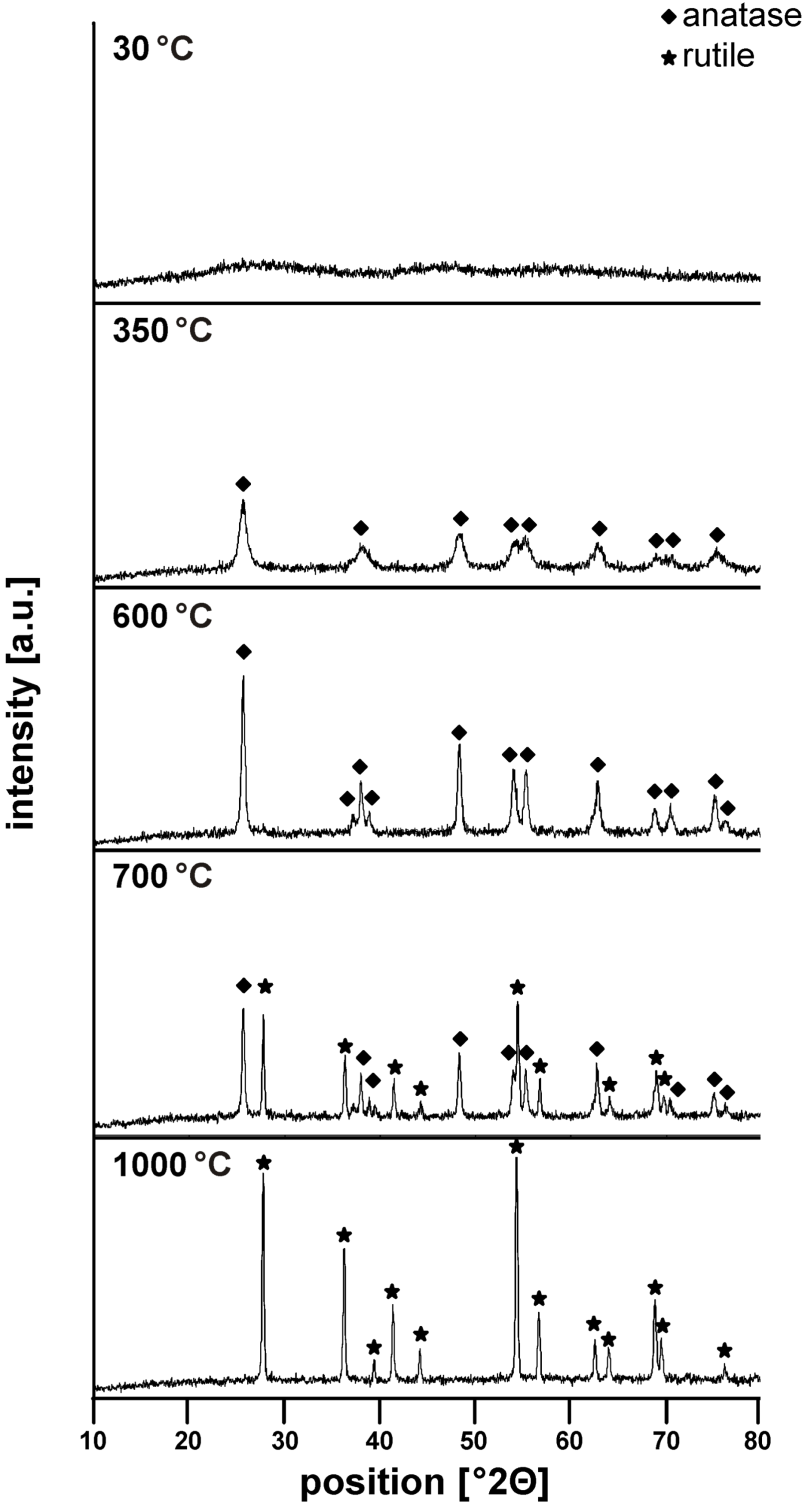
X-ray diffraction patterns obtained for TiO_2_ coatings treated at different temperatures as indicated in the figure.

The thin-film Au/TiO_2_ catalysts were prepared by an evaporation-induced self-assembly (EISA) approach, by spin-coating a Si(100) wafer with a TiO_2_ sol containing a structure-directing agent [31], followed by precipitation–deposition of Au on these films. A stable and coatable sol was only obtained at very low pH (conc. HCl), due to the high reactivity of the titanium alkoxide precursor at higher pH values. The crystallinity and morphology of the coating depends critically on the posttreatment temperature. As synthesized, the coatings possess an amorphous network structure comprising mesopores. The mesoscopic ordering of the pore system after the heat treatment was confirmed by small angle X-ray scattering, displaying a broad maximum at 2Θ = 1.35, indicating repeating unit distances of 6.54 nm (data not shown). Upon calcination, the material crystallized and anatase nanocrystallites formed at temperatures above 350 °C; crystallization was completed with increasing temperature (600 °C). Further heat treatment resulted in the formation of the thermodynamically stable polymorph rutile, with complete transformation from anatase to rutile at about 1000 °C (cf. Figure 3). Concomitantly with crystallization, the organized mesopore system collapsed during the heat treatment as expected when structure-directing agents, such as Pluronic P123, are applied [32]. Nevertheless, a porous material was obtained, built up from anatase crystallites of 9 nm diameter (calculated from the Scherrer equation) with specific surface areas (after calcination at 350 °C) of 175 m^2^·g^−1^ and a monomodal, narrow, pore-size distribution with an average pore size of 3.1 nm (see Supporting Information File 1 for experimental data).

The DP method employed for Au loading of the oxide films was not expected to cause major changes in the structure of the oxide film because of the gentle conditions, which was also confirmed in previous studies of Au/TiO_2_ catalysts based on highly dispersed mesoporous TiO_2_ supports [33].

From the ICP–OES analysis of the Au/TiO_2_ catalyst material (cast in the Petri dishes) we derived a Au content of 2.7 wt %. This is in the range of Au contents typical for realistic supported Au catalysts [25,34]. XPS measurements of the same cast material yielded a Au loading of 4.3 wt %, which is in reasonable agreement with the ICP–OES data. Contamination levels (e.g., Cl) were below the detection limit of XPS (XPS data on Au/TiO_2_ films see in the following section).

**Figure 4:**
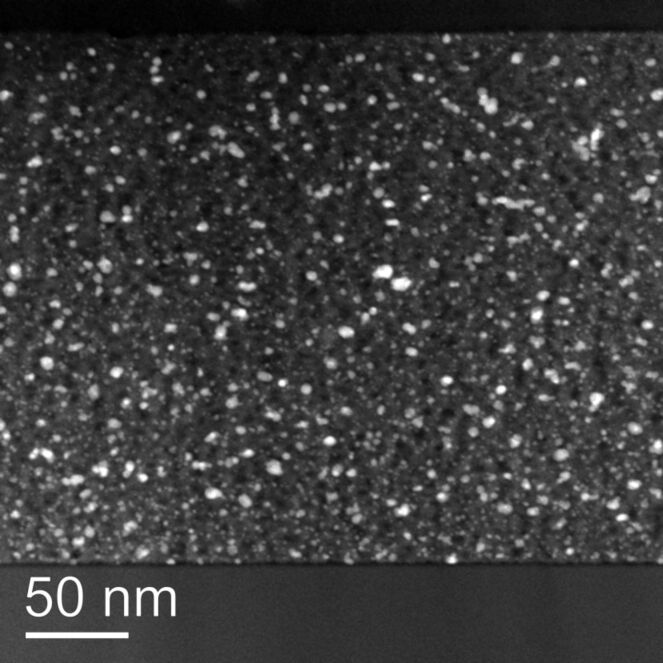
Cross-sectional scanning TEM image of a mesoporous Au/TiO_2_ film spin-coated onto a Si(100) wafer and subsequently loaded with Au.

Further information on the structural characteristics of the thin-film model catalysts was obtained from TEM analysis of the Au/TiO_2_ layers. Using the procedures described in the experimental section, cross-sectional TEM measurements were performed directly on the nanoscaled Au/TiO_2_ film, allowing for a detailed characterization of the structure of the TiO_2_ layers and of the distribution of the Au nanoparticles (NPs) in the TiO_2_ film and their size distribution. According to these measurements (see Figure 4), the mesoporous TiO_2_ films on the Si(100) substrate form a compact, homogeneous layer of polycrystalline mesoporous TiO_2_ with a uniform film thickness (~280 nm at 4000 rpm) and typical TiO_2_ crystallites of 10–20 nm. The observation of very small Au NPs agrees well with earlier findings for DP prepared Au/TiO_2_ catalysts, which generally yielded Au NPs with small sizes and a relatively uniform particle-size distribution [35].

**Figure 5:**
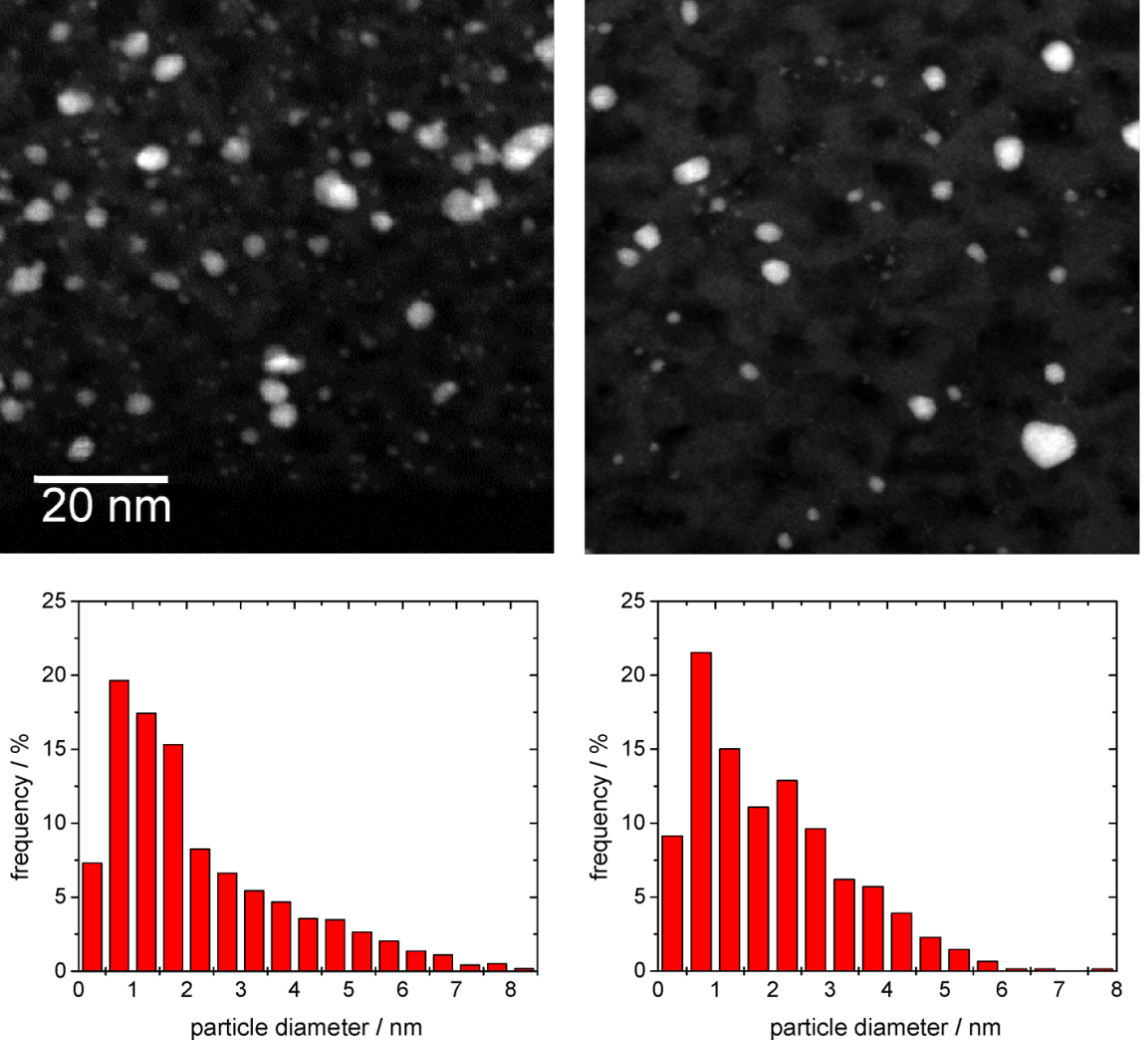
Upper part: High-magnification TEM images of the Au/TiO_2_ thin-film catalyst after oxidative pretreatment (left) and after subsequent CO oxidation reaction (300 min on stream, right); lower part: Corresponding Au particle-size distribution.

Based on the TEM analysis, the Au particles are homogenously distributed in the TiO_2_ film, with a broad particle-size distribution ranging from 0.25 to 6–8 nm. On the O350 calcined catalyst film, before CO oxidation, the maximum of the particle-size distribution is located close to ~2.0 (mean particle size 2.0 ± 1.6 nm, Figure 5, left). As expected from the much higher temperature during the calcination pretreatment, we observed no substantial changes in the gold particle-size distribution after the CO oxidation reaction (see Figure 5 right, mean particle size 2.2 ± 1.3 nm). This result closely resembles previous findings on highly dispersed Au/TiO_2_ catalysts, which also showed no significant growth of the Au NPs during reaction with similar pretreatment and reaction conditions/procedures [36–38].

Note that the size distribution of the Au nanoparticles differs significantly in the thin anatase films (280 nm thickness) compared to on the dispersed TiO_2_ supports with approximately spherical TiO_2_ particles of about 10–20 nm in diameter [33,39]. Despite the fact that we used the same preparation procedure for the Au deposition and formation of Au NPs on the TiO_2_ film as on the dispersed TiO_2_ support, the Au particles are measurably smaller than those obtained on a highly disperse mesoporous TiO_2_ support (anatase, 175 m^2^·g^−1^, maximum of the particle-size distribution at about 3.0 nm) [33,39]. Furthermore, the particle-size distribution is broader for the mesoporous TiO_2_ films. These differences may be related to differences in the Au particle growth process from a Au^3+^ solution, and specifically to the different diffusion pathways of Au^3+^ ions towards the TiO_2_ surface in the two cases, the TiO_2_ film and the TiO_2_ powder. For deposition on the TiO_2_ film samples, which were placed at the bottom of a beaker during stirring, the average diffusion path of the Au^3+^ complex to the TiO_2_ surface was larger compared to deposition on the TiO_2_ particles that were free to move in the whole volume of the solution. Consequently, the probability that an Au^3+^ ion meets the surface of a Au nanoparticle will be higher for deposition on dispersed TiO_2_ than on the TiO_2_ film. The broader Au particle-size distribution in the Au/TiO_2_ film catalyst may be due to different reasons. First, earlier TEM analysis of powder Au catalysts was performed on a Philips CM 20 instrument (200 kV, thermionic electron emission) operated in conventional bright-field TEM mode with much lower sensitivity and spatial resolution than obtained on the present instrument (FEI Titan). On the former instrument, the smallest Au particles that could be detected within the background of the porous matrix were around 1.2 nm in diameter. In the current analysis, particles with sizes down to 0.3 nm could be detected since the scanning mode of a field emission STEM using a HAADF detector delivers a very good signal-to-background ratio, especially for particles consisting of heavy elements within a matrix of low-atomic-number elements (contrast scales with approximately *Z*^2^). This may at least partly explain the higher probability of very small Au NPs in the present Au/TiO_2_ film catalysts as compared to previous data on dispersed Au/TiO_2_ catalysts. Second, although we have no direct evidence, we cannot rule out effects from residual Cl in the thin film catalysts. The presence of chloride anions is known to enhance the mobility and aggregation of Au NPs [40–41], and we cannot rule out that the residual Cl contents in the Au/TiO_2_ film catalyst after the DP process are slightly higher than in a powder Au/TiO_2_ catalyst. Even at levels far below the detection limit of XPS (~0.01 ML), Cl could have measurable effects.

### XPS results

**Figure 6:**
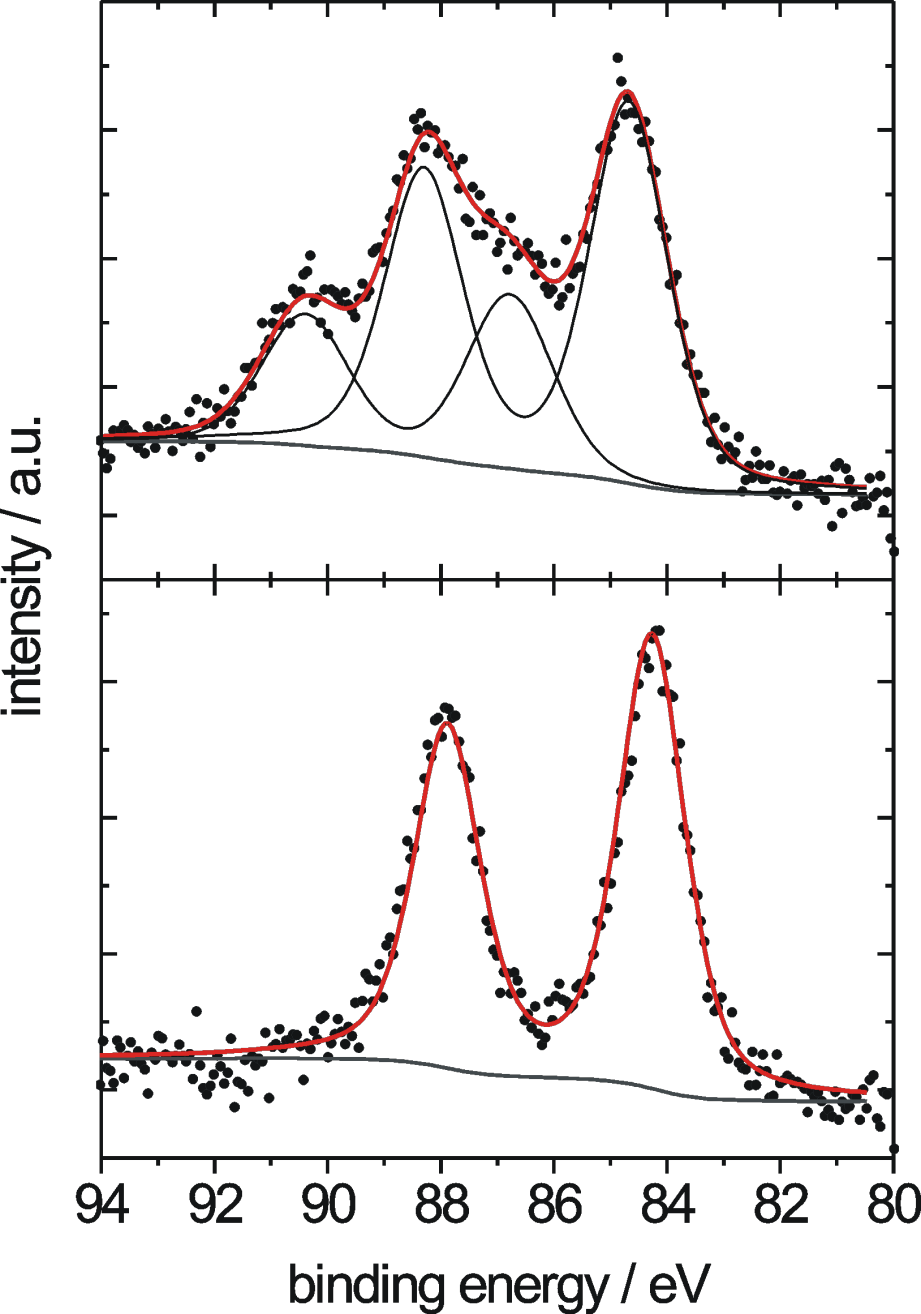
Au(4f) signals of the Au nanoparticles in mesoporous Au/TiO_2_ catalyst films before (upper panel) and after (lower panel) oxidative pretreatment.

The composition of the Au/TiO_2_ catalyst layer surface, in particular the oxidation state of the Au NPs and the amount of Au present in the film, was characterized by XPS, both before and after the oxidative pretreatment. Survey spectra showed the presence of Au, Ti, oxygen, and carbon species; significant carbon contributions are attributed to contaminations picked up during the sample transfer through air after drying or after calcination. Representative detail spectra of the Au(4f) region are displayed in Figure 6 (upper and lower panel). The Au(4f) spectrum of the dried catalyst, prior to calcination, includes two Au related contributions, a metallic Au^0^ species with a Au(4f_7/2_) signal at 84.5 eV as the main component (intensity ~66% of the total Au(4f) intensity), and a second pair of peaks related to ionic Au species [42–44]. The latter peaks appear at 1.9 eV higher binding energy compared to the metallic Au species, indicative of a Au^3+^ species [42,44]. The binding energy of the metallic Au(4f_7/2_) peak was calibrated with respect to the Ti(2p_3/2_) peak of the mesoporous TiO_2_ (*E*_B_ = 459.0 eV) [33,44]. The total Au(4f) intensity corresponds to a Au content of 8.6 (as prepared) and 5.2 (after calcination) atom %, equivalent to 41 wt % (as prepared) and 26 wt % (after calcination). The loss in Au(4f) intensity upon O350 treatment results from Au^0^ particle formation, which increases the absorption of Au(4f) electrons as compared to a dispersed distribution of Au^3+^ ions and very small Au^0^ NPs. After calcination, the Au(4f) signal only shows the spin–orbit splitting of the Au(4f) state of Au^0^ species, without any indication of ionic species.

The significantly higher Au content in the Au/TiO_2_ thin-film catalyst as compared to the dispersed Au/TiO_2_ catalyst (cf. value of 4.3 wt % in the cast material given before) may arise from the fact that XPS measurements are sensitive only to the uppermost layers (a few nanometers) of the sample surface. An inhomogeneous distribution of the Au NPs in the film, with a pronounced enrichment at or close to the film surface, would result in much higher measured Au(4f) signals than obtained for a homogeneous Au NP distribution, at identical total Au contents. The TEM results, however, clearly indicate a homogeneous distribution of the Au NPs in the film, and a similar result was also obtained from high resolution SEM measurements, which resolved a lateral distribution of Au NPs and surface sensitive conditions (at 1 keV beam energy), which is compatible with that observed in the TEM images. Finally, Au contents of 15 wt % and higher were obtained also in EDX spot measurements on Au/TiO_2_ thin-film catalysts. These probe the entire film thickness, and even into the Si substrate, as evident from the presence of a visible Si peak. Hence, despite a similar Au loading process and process parameters, the DP process leads to significantly higher Au contents on the TiO_2_ film samples than on highly disperse TiO_2_ powder. Most easily, this can be explained by the much smaller mass and surface area of the TiO_2_ films (~0.1 mg per batch with 4–5 film samples) as compared to that of the dispersed TiO_2_ support (~10 mg per batch) during Au deposition in identical solution volumes.

In total, most of the structural properties of the mesoporous TiO_2_ thin films on Si(100) substrates (crystallinity, pore size) are similar to those found in the mesoporous TiO_2_ powder. Subsequent Au loading leads to a homogeneous distribution of the Au nanoparticles with a slightly smaller mean size, but a broader size distribution than obtained for disperse TiO_2_ supports, for both mesoporous or nonporous (P25) supports. The Au content in the thin films, however, is significantly higher than in the disperse material, and must be reduced in future work. On the other hand, when comparing with typical planar Au/TiO_2_ model systems, consisting, e.g., of Au nanoparticles deposited on single-crystalline TiO_2_(110) supports by evaporation under UHV conditions, the size distributions are of comparable width. In contrast, depositing preformed Au nanoparticles, prepared by micellar techniques, yields model catalysts with much narrower size distributions and approximately spherical Au NPs of comparable size [36]. Hence, based on their structural characteristics, mesoporous Au/TiO_2_ thin film catalysts can be regarded as structurally well-defined planar model systems, which are closer to realistic catalysts than conventional planar model catalysts, but are nevertheless structurally well defined and thus are a suitable candidate to bridge the materials gap.

### Catalytic properties of the mesoporous Au/TiO_2_ films

#### CO_2_ formation as function of time

The catalytic activity of the Au/TiO_2_ films for CO oxidation was investigated in different ways. First, we tested the initial activity and the tendency for deactivation as a function of time in stream.

**Figure 7:**
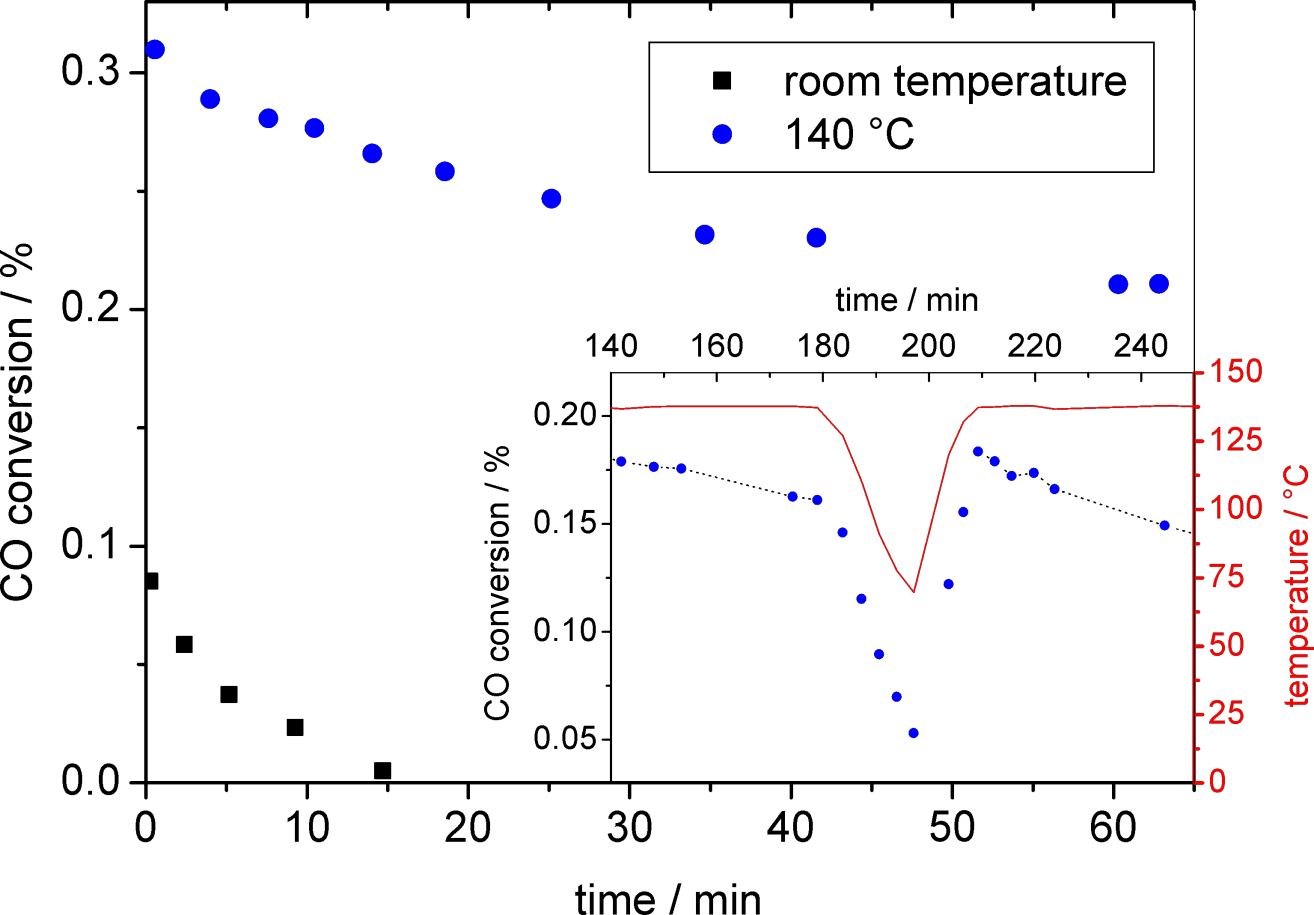
CO conversion during CO oxidation over a mesoporous Au/TiO_2_ film as a function of time and sample temperature, at room temperature and at 140 °C. The inset shows the further development of the CO conversion at 140 °C (blue dots) up to 3 h (180 min) under identical conditions and the effect of subsequently modifying the temperature (red curve). Dotted lines serve as guides for eye.

Figure 7 shows the catalytic activity of the mesoporous Au/TiO_2_ films as a function of time (CO:O_2_ = 1:1, total pressure: 2 mbar) in two different temperature regimes, at 140 °C (blue dots) and at room temperature (black squares). The data reveal a rapid deactivation of the film catalyst at room temperature (50% of initial activity value is lost within a few minutes), whereas at 140 °C the decrease in activity is much slower (50% after 3 h, see inset before temperature modulation).

Possible reasons for the deactivation of TiO_2_ supported Au catalysts are the agglomeration/sintering of Au particles (“irreversible deactivation”) and the accumulation of stable adsorbed species, such as surface carbonates or water, on the catalyst surface [23–24,38,45–46]. The latter deactivation process is reversible, if the adsorbed species can be removed without affecting the catalyst itself, e.g., by thermal desorption. Since, based on the TEM measurements, there was no significant change in the mean particle size during the reaction, Au NP sintering can be ruled out as the main reason for the rapid deactivation. Moreover, since particle agglomeration/sintering is a thermally activated process, this process should be faster at higher temperatures, which is in contrast to our observation. The much faster deactivation of the catalyst at room temperature instead points to an enhanced accumulation of stable adsorbed species, which may cause blocking of active sites [23–24,38,45–46]. At higher temperatures, the enhanced desorption/decomposition of these surface species results in slower accumulation rates and hence slower deactivation, in agreement with our findings [38]. The nature of the site-blocking adsorbate, however, is not clear from these experiments. It is likely that, similar to previous findings based on combined in situ IR and reaction measurements on dispersed Au/TiO_2_ catalysts [23], surface carbonates are mainly responsible for the deactivation [24,38,45–46]. However, additional effects from other species, e.g., residues of the synthesis process that are still present in the support material after the calcination procedures prior to the reaction measurement, cannot be ruled out. Evidence for an important role of surface carbonates in the deactivation process comes from a measurement in which the CO_2_ evolution was followed while lowering the reaction temperature from 140 °C to 70 °C and then returning to 140 °C again, where it was held for 1 h (inset of Figure 7). In that measurement, we found that the CO_2_ production after the initial decay increased to even higher values after the reheating to 140 °C than were measured before the temperature variation, that is from 50% to 60% of the initial signal intensity. Afterwards, the CO_2_ signal slowly returned to values as they would have been expected without the intermittent temperature variation. Most simply, the higher CO_2_ formation rate after the heat-up procedure can be understood by additional CO_2_ formation due to the decomposition of carbonate species that were accumulated on the surface at the previously lower temperature [46]. Once the excess surface carbonates are removed, the CO_2_ formation rate returns to its ‘normal’ value. While this argument appears plausible, definite proof for this hypothesis must wait for in situ IR measurements on the Au/TiO_2_ thin-film catalyst, which are planned for the future.

The fact that the deactivation at 140 °C is still faster than observed on a dispersed Au/TiO_2_ catalyst supported on nonporous P25 (Degussa) at the same temperature [38] may be explained by the smaller TiO_2_ surface area available per Au NP in the thin film catalysts, which results from a combination of a much higher Au loading (~25 wt % versus 3.3 wt % in [38]), a smaller Au NP size (2.0 nm versus 3.2 nm in [38]), and a higher surface area (175 m^2^·g^−1^ versus 56 m^2^·g^−1^ in [38]). In total, the surface area per Au NP decreases to below one third of the value in the Au/P25 catalysts, and therefore surface blocking by stable adsorbed reaction by-products could be correspondingly faster. Similar effects were proposed recently by van den Berg et al. for a Au/TiO_2_-MCM-48 catalyst [47]. On the other hand, the fact that there was little difference in deactivation between dispersed mesoporous and nonporous TiO_2_ supported Au/TiO_2_ catalysts, despite the much higher surface area of the mesoporous catalysts [38,48], contradicts this proposal. Furthermore, the very rapid deactivation at room temperature, which is considerably faster than observed for dispersed Au/TiO_2_ catalysts at similar reaction temperatures (not shown), indicates that in that case contributions from other site-blocking adsorbates that are not present on P25 based Au/TiO_2_ catalysts, play a role as well. In summary, the physical origin for the rapid deactivation is not yet clear, but most likely it is related to more than a single effect. Finally it should be noted that absolute rates cannot be derived from these measurements at present, and further work focusing on that aspect is in progress.

#### CO oxidation activity as a function of film thickness

**Figure 8:**
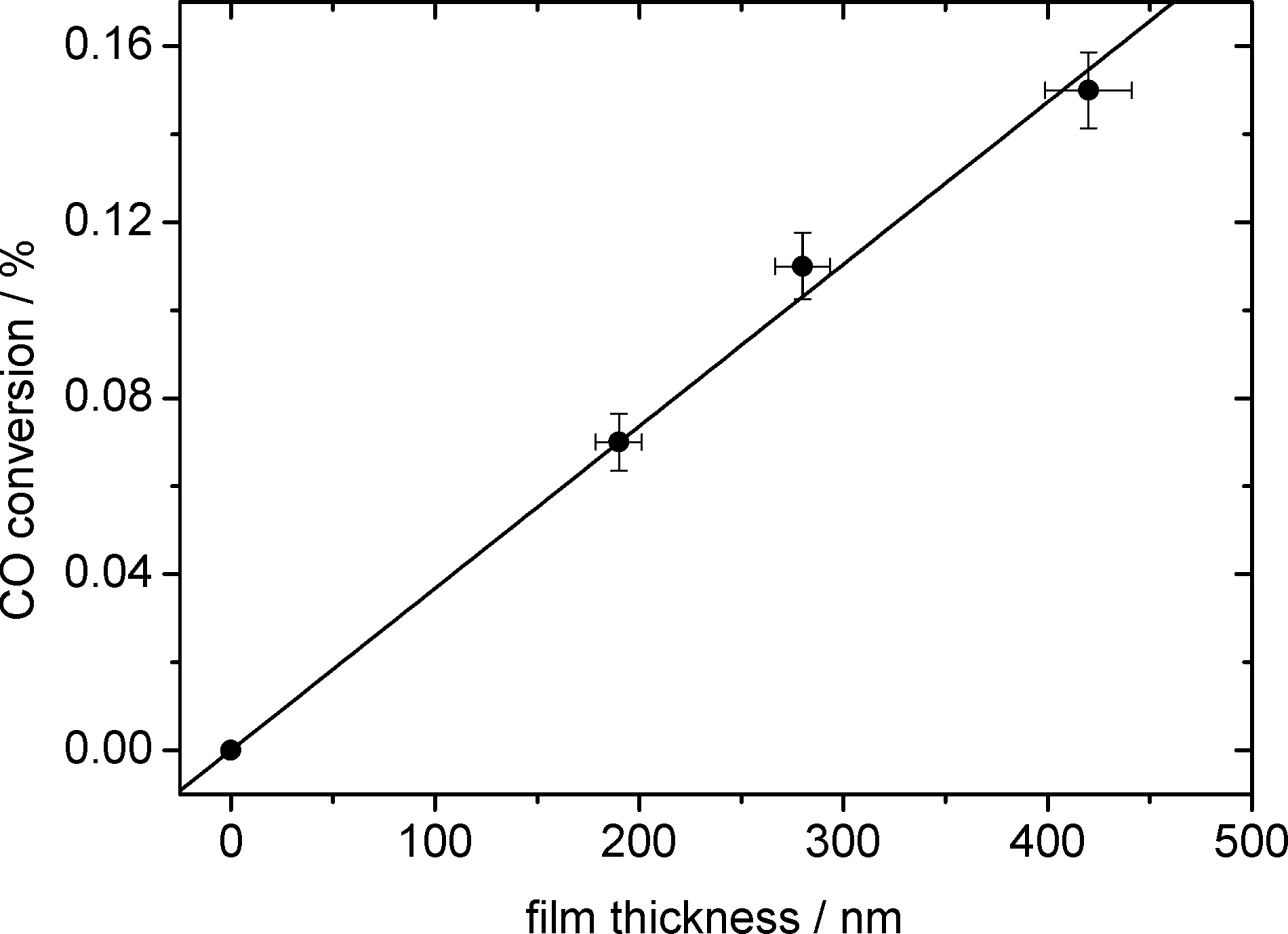
CO conversion measured above mesoporous Au/TiO_2_ films of different thicknesses.

A second important aspect in the catalytic properties of these film catalysts is related to transport effects, specifically to the accessibility of deeper lying regions within the mesoporous Au/TiO_2_ films by the reaction gases. This was investigated by measuring the activity of three samples with different film thicknesses (190 nm, 280 nm and 420 nm), which were fabricated with different rotation speeds (2000 rpm, 4000 rpm and 6000 rpm) during the spin-coating process. For direct comparison, the different TiO_2_ film samples were loaded with Au simultaneously, using the same aqueous solution of HAuCl_4_·3H_2_O. The catalytic activity of the Au/TiO_2_ film samples towards CO oxidation was measured at 140 °C in a 1:1 mixture of CO and O_2_ (total pressure 2 mbar). To reduce the effect of deactivation before or during the measurements, the samples were heated to 140 °C before adding the reactant gases, and the catalytic activity was evaluated within the first minutes after adjusting the CO and O_2_ gas flows. The resulting CO conversions, corrected for contributions from the background intensity (see Experimental section), were plotted versus the respective film thickness (Figure 8). Considering also that the background corrected CO_2_ signal (CO conversion) on the bare Si wafer (film thickness: 0 nm) must be zero by definition, we obtain a linear fit to the data with a slope corresponding to an increase of the CO conversion of 0.037% per 100 nm Au/TiO_2_ film layer.

The linear increase of the CO_2_ evolution/CO conversion with increasing film thickness is clear proof that under the given reaction conditions transport effects within the mesoporous Au/TiO_2_ film are negligible. This together with a low conversion leads to identical reaction conditions at all locations on and in the film sample. This result implies that in further catalytic reaction measurements on these film catalysts, the product gas evolution detected atop a certain position on the film surface is representative for the entire underlying layer volume, and can be normalized accordingly. Lateral transport of product molecules within the film can be neglected considering that the diameter of the sampled surface area (2.5 mm) is large in comparison to the film thickness of 200–400 nm.

#### CO oxidation: Apparent activation energy and reaction orders

A third aspect deals with inherent reaction properties such as the apparent activation energy (temperature dependence of the reaction rate) and the reaction orders (partial pressure dependence of the reaction rate). Here it is of interest whether the model systems exhibit characteristics that are comparable to those of realistic dispersed catalysts, in this case to those of dispersed Au/TiO_2_ catalysts.

**Figure 9:**
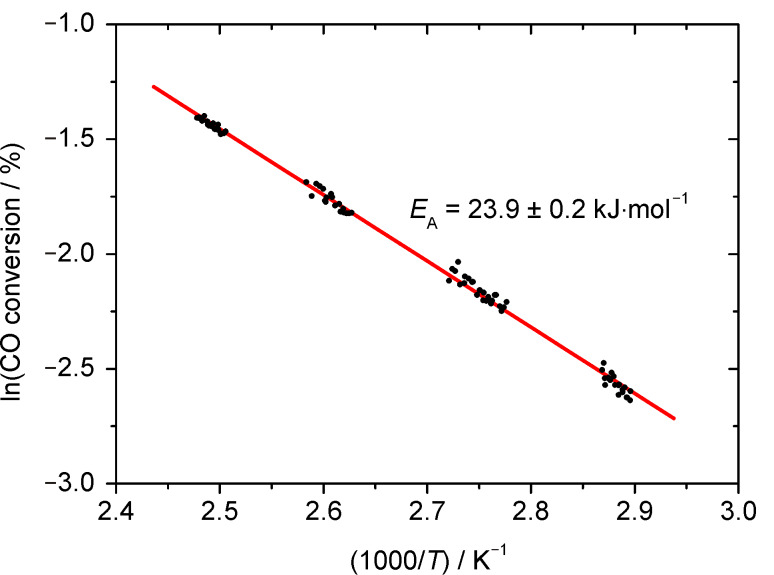
Arrhenius plot of the CO conversion, which is proportional to the CO oxidation rate, to determine the apparent activation energy *E*_A_ for Au nanoparticles in a mesoporous TiO_2_ film.

The apparent activation energy *E*_A_ was determined on a 280 nm thick Au/TiO_2_ film by varying the temperature during the measurement between 70 and 130 °C while recording the CO_2_ production (CO:O_2_ = 1:1, total pressure 2 mbar). In order to reduce the impact of contributions from catalyst deactivation (see above), we started with the high temperatures. The resulting logarithmic CO conversions are plotted versus the inverse temperature in Figure 9. The gaps in between the four groups of data points result from measurements on the reference sample (cf. Figure 2). The apparent activation energy of *E*_A_ = 23.9 ± 0.2 kJ·mol^−1^, obtained from the Arrhenius plot, is comparable in size to results obtained under similar reaction conditions on other model systems (Diemant et al. [49]: 27 kJ·mol^−1^, Valden et al. [50]: 15–23 kJ·mol^−1^) or on dispersed catalysts (Bollinger et al. [51]: 29 kJ·mol^−1^, Liu et al. [52]: 24 kJ·mol^−1^, Haruta et al. [35]: 34 kJ·mol^−1^, and Schumacher et al. [53]: 27 kJ·mol^−1^). Considering the still-existing differences in reaction conditions (reaction gas pressure and composition, catalyst pretreatment), the numbers indicate a good agreement in the reaction characteristics of the Au/TiO_2_ film catalysts and the realistic Au/TiO_2_ catalysts.

**Figure 10:**
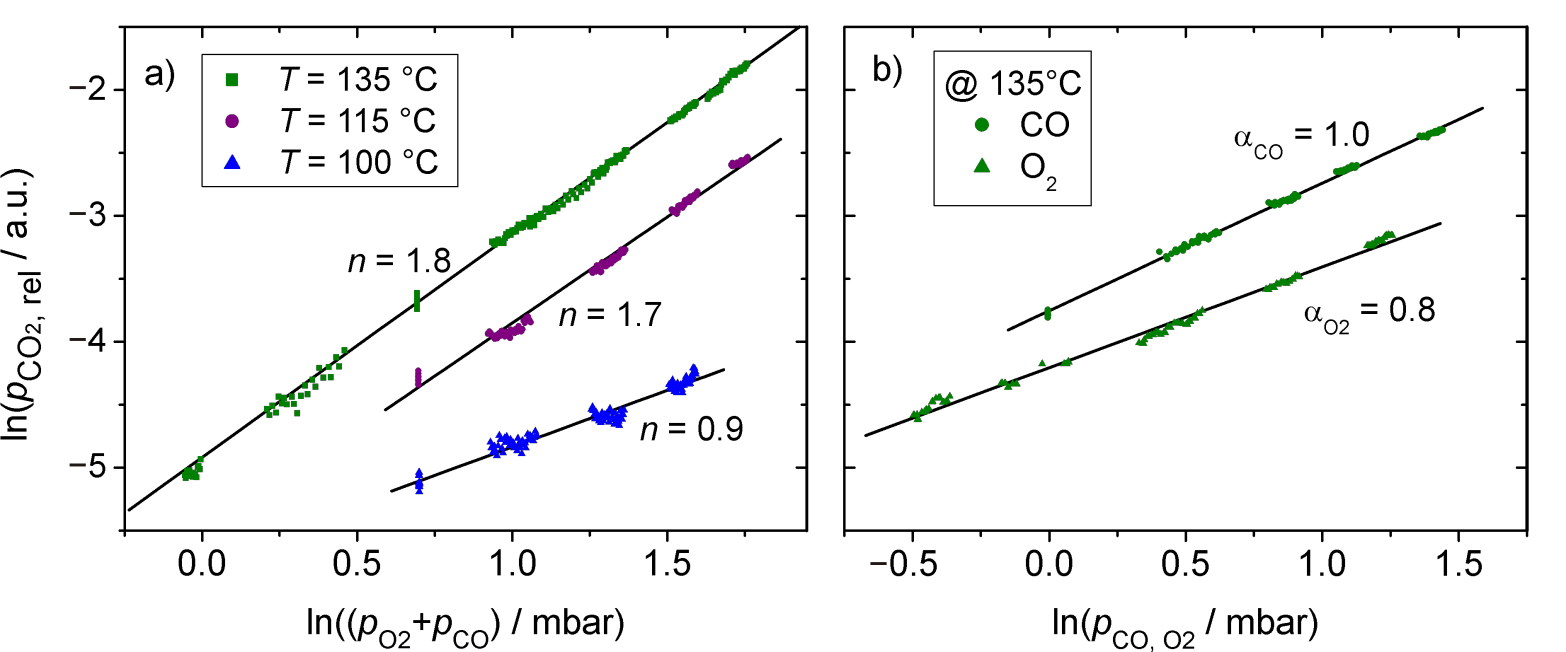
a) Determination of the total reaction order *n* in the CO oxidation reaction at temperatures of 100 °C, 115 °C and 135 °C by varying the total pressure from 0.5 to 5 mbar at constant reaction gas composition (CO:O_2_ = 1:1). b) Partial reaction orders of O_2_ and CO at a reaction temperature of 135 °C and constant partial pressure of the other reactant (1 mbar).

Further information on the reaction characteristics comes from the reaction orders, i.e., the pressure dependence of the reaction rate *r*_CO2_ in a power law rate description:

[1]
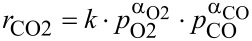


with the total reaction order *n* being the sum of the partial reaction orders α_CO_ and α_O2_. In order to determine the total reaction order *n*, we performed a series of measurements in which we varied the total pressure in the reaction chamber between 0.5 and 5 mbar, while keeping the composition of the gas mixture constant at CO:O_2_ = 1:1. Similar measurements were performed for three different temperatures, 100 °C, 115 °C and 135 °C. For reaction at 135 °C, the partial reaction orders α_O2_ and α_CO_ were additionally determined by varying either the CO (*p*_CO_) or the O_2_ partial pressure (*p*_O2_), while keeping the other component constant (1 mbar). The resulting data were evaluated according to Equation 1, by plotting the logarithmic CO conversion versus the logarithmic total pressure (total reaction order *n*, Figure 10a) or versus the logarithmic CO or O_2_ partial pressure (partial reaction orders α_O2_ and α_CO_, Figure 10b).

The calculated total reaction orders *n* vary between 0.9 for 100 °C and 1.7–1.8 for 115 °C and 135 °C. The reaction order at *T* = 100 °C obtained in these experiments (*n* = 0.9 ± 0.1) is in good agreement with results from our group on model systems (Diemant et al.: *n* = 0.88 [49]). Comparison with corresponding reaction data for dispersed, realistic, Au/TiO_2_ catalysts is limited by large differences in the reaction conditions, in particular in the composition of the reaction gas, but including also the total pressure, and the reaction temperature. Accordingly, typical total reaction orders measured on Au/TiO_2_ powder catalysts were found to vary between *n* = 0.3 and 2.1 (e.g., Haruta et al. [35]: *n* = 0.3; Bollinger et al. [51], Liu et al. [52], Cant et al. [54]: *n* = 0.65; Lin et al. [55]: *n* = 0.9; Bondzie et al. [56]: *n* = 2.1), an overview is given in [34]. In addition to the reaction gas composition, also the preparation method and the pretreatment of the catalyst may affect these values. Concentrating on reaction conditions comparable to the present measurements, Schumacher et al. reported a total reaction order of *n* = 1.11 for reaction at 80 °C [53]. Considering the trend in our experimental data, which reveals a pronounced decrease of the reaction order with decreasing temperature, we expect a value of below *n* = 0.8 for the total reaction order at 80 °C, which fits well with the above numbers.

Comparison of the partial reaction orders in these measurements and those reported in previous studies equally suffers from the variation in reaction conditions. Nevertheless, we can summarize a few of the characteristics which seem to be valid over a large range of reaction conditions. For instance, in most studies (except for that by Liu et al. [52]), the reaction order of CO was larger than that for O_2_, and it was always positive. The latter finding, which is in contrast to observations on platinum metal catalysts in the low-rate branch, indicates that the reaction is not limited by CO_ad_-induced blocking of active sites, but by a lack of CO_ad_. The partial reaction orders determined in these experiments at 135 °C (cf. Figure 10a) follow these trends, the CO reaction order (α_CO_ = 1.0) is higher than the corresponding O_2_ value (α_O2_ = 0.8). This is also compatible with the previous observation that at 1 mbar and 140 °C the steady-state CO_ad_ coverage is very low already in the absence of O_2_, due to rapid CO_ad_ desorption, and is even less in the presence of O_2_ [38]. For CO oxidation on dispersed Au/TiO_2_ catalysts, CO and O_2_ reaction orders of *n* = 0.34 (at 1 mbar O_2_, CO variable) and *n* = 0.32 [24] or 0.38 [57], respectively (at 1 mbar CO, O_2_ variable), were reported for reaction at 80 °C and comparable gas-phase compositions. The much lower reaction orders in the above two studies can be explained by the lower reaction temperatures in those cases. Considering the steep increase of the total reaction order with temperature, by almost 100% upon increasing from 90 °C to 135 °C, we would expect a similar effect also for the partial reaction orders. The resulting values of around *n* = 0.4 for the partial reaction orders would agree perfectly with the findings in the earlier studies.

In summary, we demonstrated that the local catalytic properties of these nanoscaled mesoporous Au/TiO_2_ films largely resemble those of commonly investigated Au/TiO_2_ catalysts supported by highly disperse, nonporous or mesoporous TiO_2_. This is a precondition for their use as a model catalyst. Future research will concentrate on i) clarifying the physical origin of the faster deactivation at room temperature, and in particular on ii) exploring the role of transport effects in these films on the reaction characteristics, both by locally resolved measurements on microstructured samples by means of higher resolution scanning mass spectrometry and by time-resolved measurements, e.g., in a temporal analysis of products reactor.

## Conclusion

Aiming at model catalyst systems with close-to-realistic internal transport properties**,** we have prepared nanoscaled mesoporous Au/TiO_2_ films of 200–400 nm thickness, with Au nanoparticles embedded in a mesoporous TiO_2_ film, and investigated their structural, chemical and catalytic properties. The systems were prepared by spin-coating of a mesoporous TiO_2_ film from solutions of ethanolic titanium tetraisopropoxide and Pluronic P123 on planar Si(100) substrates, calcination at 350 °C and subsequent Au loading by a deposition-precipitation procedure, followed by a final calcination step for catalyst activation. N_2_ adsorption and XRD measurements revealed a surface area of 175 m^2^·g^−1^ and a repeat unit of ~6.5 of the mesostructured anatase TiO_2_ films after calcination. After Au loading, XPS and EDX measurements determined Au contents of between 15 and 30 wt %, which is much higher than obtained by the same DP procedure on otherwise similar highly disperse TiO_2_ material (cast material, 3–4 wt %). After calcination, only metallic Au^0^ was detected in the film, which was present as Au NPs, in agreement with previous findings for dispersed Au/TiO_2_ catalysts. Cross-sectional TEM measurements revealed a homogeneous distribution of very small Au nanoparticles in the TiO_2_ film, with a maximum in the size distribution at 2.0 nm; these findings were supported also by highly surface sensitive SEM images (no enrichment of Au NPs at the film surface). Reaction measurements of the CO oxidation reaction, performed with a scanning mass spectrometer directly above the film, yielded reaction characteristics that are very close to those of highly dispersed Au/TiO_2_ catalysts at comparable reaction conditions, with an activation energy of 23.9 kJ·mol^−1^ and temperature-dependent positive reaction orders (at 135 °C α_CO_ ≈ 1.0 and α_O2_ ≈ 0.8). Furthermore, the observation of a linear increase of the activity with increasing film thickness indicates that mass transport limitations inside the film are essentially absent (below the detection limit), and the reaction conditions (partial pressures) inside the film are independent of the location.

The good agreement between the results presented here and those for dispersed Au/TiO_2_ powder catalysts illustrates that this model system is well suited to further bridge the materials gap between model studies and real catalysts. The absence of transport limitations in the Au/TiO_2_ films, as evidenced by the linear increase of activity with thickness, together with the ability of locally resolved mass spectrometric measurements will allow us to investigate possible transport effects. Accordingly, future work will include studies of microstructured Au/TiO_2_ film patterns for investigating transport effects, and more detailed studies of structural effects imposed, e.g., by changes in the film morphology or in the Au particle size, and finally such work will focus on establishing in situ spectroscopy techniques.

## Supporting Information

Supporting Information features details on the nitrogen sorption measurement of porous titania.

File 1 Details of sorption measurements.

## References

[1] Sinfelt J H (2002). Surf Sci.

[2] Stoltze P, Nørskov J K (1985). Phys Rev Lett.

[3] Aßmann J, Löffler E, Birkner A, Muhler M (2003). Catal Today.

[4] Over H, Muhler M (2003). Prog Surf Sci.

[5] Imbihl R, Behm R J, Schloegl R (2007). Phys Chem Chem Phys.

[6] Vang R T, Lægsgaard E, Besenbacher F (2007). Phys Chem Chem Phys.

[7] Ertl G (2008). Angew Chem, Int Ed.

[8] Rasmussen P B, Hendriksen B L M, Zeijlemaker H, Ficke H G, Frenken J W M (1998). Rev Sci Instrum.

[9] Kolmakov A, Goodman D W (2003). Rev Sci Instrum.

[10] Tao F, Tang D, Salmeron M, Somorjai G A (2008). Rev Sci Instrum.

[11] Pantförder J, Pöllmann S, Zhu J F, Borgmann D, Denecke R, Steinrück H-P (2005). Rev Sci Instrum.

[12] Salmeron M, Schlögl R (2008). Surf Sci Rep.

[13] Ogletree F D, Bluhm H, Hebenstreit E D, Salmeron M (2009). Nucl Instrum Methods Phys Res, Sect A.

[14] Knop-Gericke A, Kleimenov E, Hävecker M, Blume R, Teschner D, Zafeiratos S, Schlögl R, Bukhtiyarov V I, Kaichev V V, Prosvirin I P (2009). Adv Catal.

[15] Beitel G A, Laskov A, Oosterbeek H, Kuipers E W (1996). J Phys Chem.

[16] Zhao Z, Diemant T, Häring T, Rauscher H, Behm R J (2005). Rev Sci Instrum.

[17] Dupont C, Loffreda D, Delbecq F, Santos Aires F J C, Ehret E, Jugnet Y (2008). J Phys Chem C.

[18] Weckhuysen B M (2004). In-situ Spectroscopy of Catalysts.

[19] Singh J, Lamberti C, van Bokhoven J A (2010). Chem Soc Rev.

[20] Rainer D R, Goodman D W (1997). J Mol Catal A.

[21] Kielbassa S, Kinne M, Behm R J (2004). J Phys Chem B.

[22] Freund H-J, Pacchioni G (2008). Chem Soc Rev.

[23] Schumacher B, Plzak V, Kinne M, Behm R J (2003). Catal Lett.

[24] Schumacher B, Denkwitz Y, Plzak V, Kinne M, Behm R J (2004). J Catal.

[25] Schumacher B, Plzak V, Cai J, Behm R J (2005). Catal Lett.

[26] Brunauer S, Emmett P H, Teller E (1938). J Am Chem Soc.

[27] Barrett E P, Joyner L G, Halenda P P (1951). J Am Chem Soc.

[28] Roos M, Kielbassa S, Schirling C, Häring T, Bansmann J, Behm R J (2007). Rev Sci Instrum.

[29] Roos M, Bansmann J, Zhang D, Deutschmann O, Behm R J (2010). J Chem Phys.

[R30] 30*Partial Pressure Measurements in Vacuum Technology;* Inficon AG: Balzers, Liechtenstein, 2001.

[31] Frindell K L, Bartl M H, Popitsch A, Stucky G D (2002). Angew Chem, Int Ed.

[32] Thomas A, Schlaad H, Smarsly B, Antonietti M (2003). Langmuir.

[33] Geserick J, Fröschl T, Hüsing N, Kucerova G, Makosch M, Diemant T, Eckle S, Behm R J (2011). Dalton Trans.

[34] Aguilar-Guerrero V, Gates B C (2009). Catal Lett.

[35] Haruta M, Tsubota S, Kobayashi T, Kageyama H, Genet M J, Delmon B (1993). J Catal.

[36] Kielbassa S, Häbich A, Schnaidt J, Bansmann J, Weigl F, Boyen H-G, Ziemann P, Behm R J (2006). Langmuir.

[37] Comotti M, Li W-C, Spliethoff B, Schüth F (2006). J Am Chem Soc.

[38] Denkwitz Y, Schumacher B, Kučerová G, Behm R J (2009). J Catal.

[39] Denkwitz Y, Makosch M, Geserick J, Hörmann U, Selve S, Kaiser U, Hüsing N, Behm R J (2009). Appl Catal, B.

[40] Bowker M, Nuhu A, Soares J (2007). Catal Today.

[41] Carabineiro S A C, Silva A M T, Dražić G, Tavares P B, Figueiredo J L (2010). Catal Today.

[42] Dickinson T, Povey A F, Sherwood P M A (1975). J Chem Soc, Faraday Trans 1.

[43] Denkwitz Y, Zhao Z, Hörmann U, Kaiser U, Plzak V, Behm R J (2007). J Catal.

[44] Holm R, Storp S (1976). Appl Phys.

[45] Konova P, Naydenov A, Venkov C, Mehandjiev D, Andreeva D, Tabakova T (2004). J Mol Catal A.

[46] Clark J C, Dai S, Overbury S H (2007). Catal Today.

[47] van den Berg M W E, De Toni A, Bandyopadhyay M, Gies H, Grünert W (2011). Appl Catal, A.

[48] Denkwitz Y, Geserick J, Hörmann U, Plzak V, Kaiser U, Hüsing N, Behm R J (2007). Catal Lett.

[49] Diemant T (2008). Adsorptions- und Reaktionseigenschaften planarer PtRu/Ru(0001)- und Au/TiO2/Ru(0001)-Modellkatalysatoren: Von der Oberflächenchemie zur Katalyse.

[50] Valden M, Pak S, Lai X, Goodman D W (1998). Catal Lett.

[51] Bollinger M A, Vannice M A (1996). Appl Catal, B.

[52] Liu H, Kozlov A I, Kozlova A P, Shido T, Asakura K, Iwasawa Y (1999). J Catal.

[53] Schumacher B (2005). Investigation of the CO Oxidation on highly disperse Au/TiO2 Catalysts in H2-rich and H2-free Atmosphere – A kinetic and mechanistic Study.

[54] Cant N W, Ossipoff N J (1997). Catal Today.

[55] Lin S D, Bollinger M, Vannice M A (1993). Catal Lett.

[56] Bondzie V A, Parker S C, Campbell C T (1999). Catal Lett.

[57] Widmann D, Behm R J (2011). Angew Chem, Int Ed.

