# Supplementary material for: Nanostructured, mesoporous Au/TiO2 model catalysts – structure, stability and catalytic properties
Source: Beilstein J Nanotechnol. 2011 Sep 15;2:593–606. doi: 10.3762/bjnano.2.63 (PMC3190629; doi:10.3762/bjnano.2.63)
Supplement: File 1 — Details of sorption measurements. [file Beilstein_J_Nanotechnol-02-593-s001.pdf]

Nanostructured, mesoporous Au/TiO_2 model catalysts – structure, stability and catalytic properties

Matthias Roos[1], Dominique Böcking[2], Kwabena Offeh Gyimah[1], Gabriela Kucerova[1], Joachim Bansmann[1], Johannes Biskupek[4], Ute Kaiser[4], Nicola Hüsing[3] and R. Jürgen Behm*[1]

Address: [1]Institute of Surface Chemistry and Catalysis, Ulm University, D-89069 Ulm, Germany; [2]Institute of Inorganic Chemistry, Ulm University, D-89069 Ulm, Germany; [3]Materials Chemistry, Paris-Lodron University Salzburg, Austria and [4]Transmission Electron Microscopy Group, Ulm University, D-89069 Ulm, Germany

Email: R. Jürgen Behm* - juergen.behm@uni-ulm.de

* Corresponding author

Details of sorption measurements

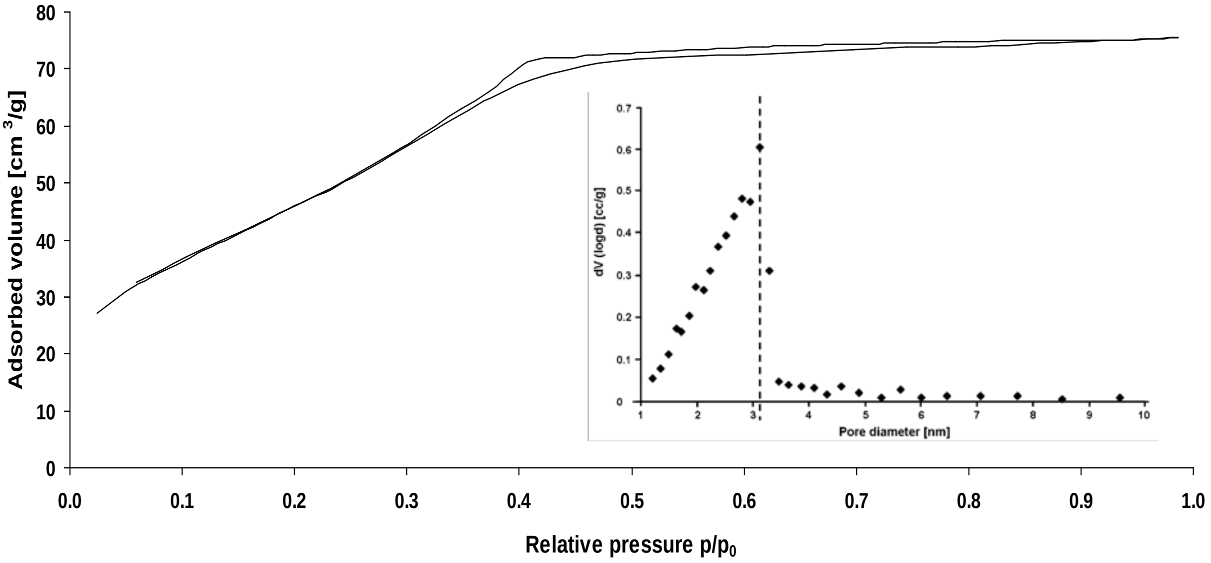

Figure S1: Nitrogen sorption measurement of porous titania. The inset shows the pore size distribution evaluated by the BJH method from the desorption branch of the isotherm.
